# Cryptic BCR-ABL fusion gene as variant rearrangement in chronic myeloid leukemia: molecular cytogenetic characterization and influence on TKIs therapy

**DOI:** 10.18632/oncotarget.15369

**Published:** 2017-02-16

**Authors:** Simona Luatti, Carmen Baldazzi, Giulia Marzocchi, Gaia Ameli, Maria Teresa Bochicchio, Simona Soverini, Fausto Castagnetti, Mario Tiribelli, Gabriele Gugliotta, Giovanni Martinelli, Michele Baccarani, Michele Cavo, Gianantonio Rosti, Nicoletta Testoni

**Affiliations:** ^1^ Department of Experimental, Diagnostic and Specialty Medicine, Institute of Hematology “L. and A. Seràgnoli”, University of Bologna, “S Orsola-Malpighi” University Hospital, Bologna, Italy; ^2^ Division of Hematology and Bone Marrow Transplantation, Azienda Ospedaliero-Universitaria di Udine, Udine, Italy

**Keywords:** CML, BCR/ABL, Philadelphia chromosome, FISH, TKI

## Abstract

At diagnosis, about 5% of Chronic Myeloid Leukemia (CML) patients lacks Philadelphia chromosome (Ph), despite the presence of the BCR/ABL rearrangement. Two mechanisms have been proposed about the occurrence of this rearrangement: the first one is a cryptic insertion between chromosomes 9 and 22; the second one involves two sequential translocations: a classic t(9;22) followed by a reverse translocation, which reconstitutes the normal morphology of the partner chromosomes. Out of 398 newly diagnosed CML patients, we selected 12 Ph-negative cases. Six Ph-negative patients treated with tyrosine kinase inhibitors (TKIs) were characterized, in order to study the mechanisms leading to the rearrangement and the eventual correlation with prognosis in treatment with TKIs. FISH analysis revealed cryptic insertion in 5 patients and classic translocation in the last one. In more detail, we observed 4 different patterns of rearrangement, suggesting high genetic heterogeneity of these patients. In our cases, the BCR/ABL rearrangement mapped more frequently on 9q34 region than on 22q11 region, in contrast to previous reports. Four patients, with low Sokal risk, achieved Complete Cytogenetic Response and/or Major Molecular Response after TKIs therapy. Therapy resistance was observed in one patient with duplication of BCR/ABL rearrangement and in another one with high risk. Even if the number patient is inevitably low, we can confirm that the rare Ph-negative CML patients do not constitute a “warning” category, meanwhile the presence of further cytogenetic abnormalities remains an adverse prognostic factor even in TKI era.

## INTRODUCTION

Chronic Myeloid Leukemia (CML) is a myelopro- liferative disorder characterized by the presence of the Philadelphia (Ph) chromosome, produced by the reciprocal translocation t(9;22)(q34;q11) [1, 2]. The resulting chimeric BCR/ABL fusion gene encodes for constitutively active tyrosine kinase protein [3]. A small amount of patients (about 5%), displaying typical features of CML, lacks the Ph chromosome by chromosome banding analysis (CBA), meanwhile shows BCR/ABL rearrangement. These cases are reported as Ph-negative (Ph-neg) CML cases and can be identified by fluorescence in situ hybridization (FISH) or by molecular analysis [4].

Two mechanisms have been proposed to explain how this kind of rearrangement can arise: the first one could be a cryptic insertion between chromosomes 9 and 22; the second mechanism could involve two sequential translocations, in which a standard t(9;22) translocation is followed by a reverse translocation with different breakpoints, that reconstitutes the normal morphology of the partner chromosomes. FISH analysis on metaphases shows the fusion BCR/ABL gene on derivative chromosome 22 (der(22)) much more frequently than on derivative chromosome 9 (der(9)) [5].

In the past, some authors reported that this kind of rearrangement was associated with worse prognosis than the classic translocation, when the patients were treated with conventional chemotherapy and/or α-interferon (α-IFN) therapy; while others did not find any differences in terms of outcome [6–8]. Moreover to our knowledge, only 9 Ph-neg CML cases treated with imatinib have been described in 5 previous reports [5, 9–12]. The outcome of 2 patients has not been clearly reported. Two patients achieved at least Major Cytogenetic Response (CgR) after imatinib treatment [9, 11–12], meanwhile 5 patients failed the treatment, suggesting that they were more resistant to imatinib therapy [5, 10]. Among the latter, a recent report [10] described two cases with fusion genes on both the homologous chromosomes 9 (Table 1).

**Table 1:** Ph-neg cases treated with imatinb and outcome reported in literature

Case	Sex/Age (years)	Pattern FISH	del(9q)	Location of BCR/ABL rearrangement	Mechanism of rearrangement	Therapy	Cytogenetic response	Reference
1	unknown	1R2G1F	NO	der(9)	Insertion on der(9)	imatinib	NR	Haigh S. et al, 2004 [5]
2	unknown	2R1G1F	NO	der(22)	Insertion on der(22)	imatinib	NR	Haigh S. et al, 2004 [5]
3	unknown	2R1G1F	NO	der(9)	2-step mechanism	imatinib	NR	Haigh S. et al, 2004 [5]
4	M/62	1R2G1F1R2G2F (trisomy 9)	unknown	der(9)	2-step mechanism	imatinib	CCgR	Fugazza G. et al, 2005 [9]
5	M/27	1R2G1F (80%)2G2F (20%)	NO	der(9)	Insertion on der(9)	hydroxyureaImatinib	NR	Brahmbhatt M. et al, 2014 [10]
6	M/30	1R2G1F	NO	der(9)	Insertion on der(9)	hydroxyureaImatinib	NR	Brahmbhatt M. et al, 2014 [10]
7	F/47	1R2G1F	NO	der(9)	Insertion on der(9)	hydroxyurea + α-IFNImatinibSCT	unknown	Batista A.S. et al, 2005 [11]
8	F/31	1R1G1F	YES	der(22)	Insertion on der(22)	hydroxyureaSCTImatinib	unknown	Batista A.S. et al, 2005 [11]
9	M/27	1R2G1F1R1G2F	YESNO	der(9)der(9)+der(22)	Insertion on der(9)2-step mechanism	Imatinib	MCgR	Bennour A. et al, 2011 [12]

Abbreviations: R=red signal; G=green signal; F=fusion signal; der(9)= derivative of chromosome 9; der(22)= derivative of chromosome 22; SCT= stem cell transplantation; NR= no response; CCgR= Complete Cytogenetic Response; MCgR= Major Cytogenetic Response.

We report an analysis of the clinical, cytogenetic and molecular characteristics of 6 patients with Ph-neg CML, treated with tyrosine kinase inhibitors (TKIs).

## RESULTS

At diagnosis, we have analysed 398 CML cases, of which 12 (3%) showed normal karyotype by CBA and BCR/ABL rearrangement by RT-PCR analysis. Of these, only 6 recently diagnosed patients were valuable for CBA, FISH and molecular analysis during TKIs therapy.

Detailed baseline genetic characteristics of the patients are presented in Table 2. RT-PCR analysis of the chimeric BCR/ABL transcript showed b_3_a_2_ transcript in 5 patients and b_2_a_2_ in only 1 patient (n.2). In 4 evaluable patients, no mutation in ABL gene was found at diagnosis and during the following treatment.

**Table 2:** FISH and molecular features of the Ph-neg patients

Patients	FISH signal pattern^a^	Location of BCR/ABL fusion gene	Molecular transcript	Mutational status
1	1R1G1F	chr. 9	b_3_a_2_	unknown
2	1R2G1F	chr. 9	b_2_a_2_	wild-type
3	1R2G1F	chr. 9	b_3_a_2_	unknown
4	1R2G1F	chr. 9	b_3_a_2_	wild-type
5	2R1G1F	chr. 22	b_3_a_2_	wild-type
6	1R1G2F	chr. 9 and 22	b_3_a_2_	wild-type

^a^Using Vysis LSI BCR/ABL dual-color, dual fusion FISH probe system: R=red signal, G=green signal, F=fusion signal

Dual-Color Dual Fusion (DCDF) FISH analysis revealed BCR/ABL fusion signal on der(9) in 4 patients (n.1-4), on der(22) in 1 patient (n.5) and on both derivative chromosomes in the last one (n.6).

**Figure 1:**
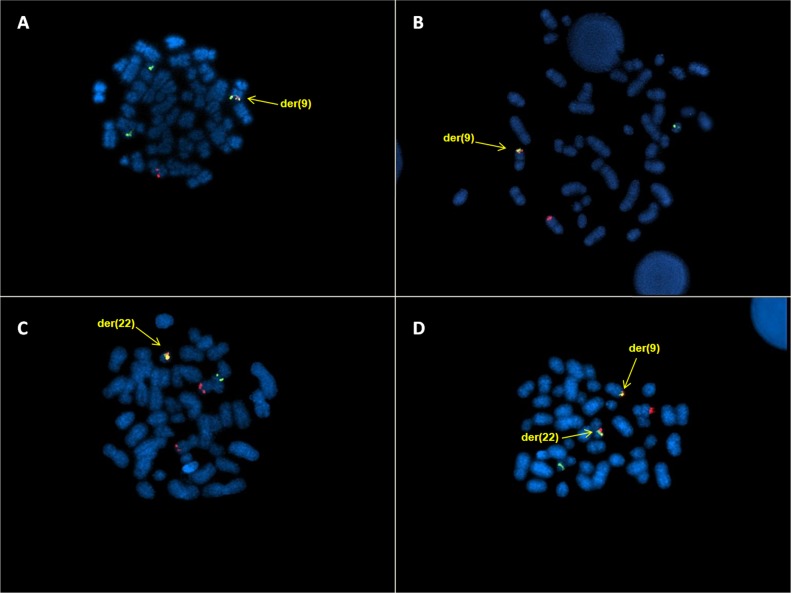
DCDF FISH analysis showing: **A**. 1R2G1F signal pattern and the BCR/ABL fusion gene on der(9); **B**. 1R1G1F signal pattern and the BCR/ABL fusion gene on der(9) **C**. 2R1G1F signal pattern and the BCR/ABL fusion gene on der(22); **D**. 1R1G2F signal pattern and the BCR/ABL fusion genes on der(9) and der(22).

Three patients (n.2-4), beyond BCR/ABL rearrangement on der(9), revealed a red ABL signal and a green BCR signal on the normal chromosomes and a smaller BCR signal on der(22), as consequence of the insertion of the BCR region into ABL region (Figure 1A). The second smaller BCR signal was not observed in 1 patient (n.1) (Figure 1B). The only patient (n.5) with BCR/ABL fusion signal on der(22) showed BCR signal on the normal chromosome 22 and ABL signals on both chromosome 9 (Figure 1C). The classic FISH pattern (2 fusion, 1 ABL and 1 BCR signals) was observed in the last patient (n.6, Figure 1D). Therefore, 4 different FISH signal patterns were observed in 6 patients.

By Tri-Color Dual Fusion (TCDF) FISH analysis, the rearrangement was confirmed as insertion of the BCR region into the ABL region in the patients with BCR/ABL fusion on der(9) (n.1-4) and insertion of the ABL region into the BCR region in the patient with BCR/ABL fusion on der(22) (n.5). The classic pattern of the last case (n.6) could be explained with 2-step mechanism: the first translocation involved the classic breakpoints and the second translocation with different breakpoints reconstituted the affected chromosomes without modifying the fusion gene.

**Figure 2:**
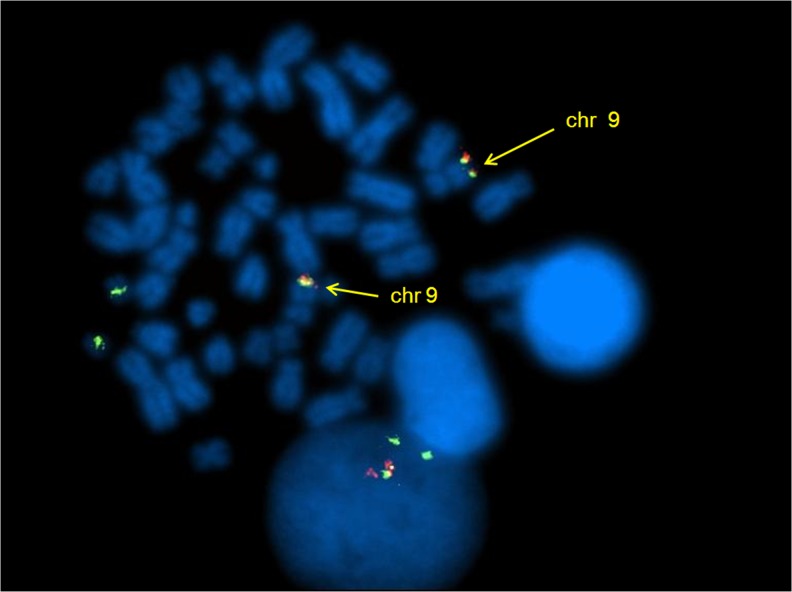
DCDF FISH analysis showing 1R1G2F signal pattern and the BCR/ABL fusion genes on both chromosomes 9

Moreover, 2 patients (n.1 and 3) (33,3%) harbored deletions adjacent to the BCR and/or ABL breakpoints: on both der(9) and der(22) chromosomes in patient n.1 and on der(9) in patient n.3. During the disease progression of the patient n.2, FISH analysis revealed the appearance of a second clone characterized by two fusion BCR/ABL and two BCR signals, that gradually replaced the first clone with only one fusion signal. The clonal evolution was concomitant with the increase of transcript detected by RQ-PCR. By FISH analysis on metaphase, the 2 fusion signals were observed on both chromosomes 9 (Figure 2), suggesting the duplication of BCR/ABL rearrangement, as sign of clonal evolution.

During imatinib therapy, interphase-FISH analysis revealed decrease of BCR/ABL positive cells in 3 patients (n.1, 3 and 5) and after a median time of 24 months (range 18-30 months) they reached the Complete CgR (CCgR) by FISH, while two patients (n.2 and 6) retained high rate of BCR/ABL positive cells by FISH (81,5% and 79%, respectively). Patient n.2 stopped imatinib because of the appearance of intestinal malignancy and later he died. Patient n.6 failed imatinib therapy, started nilotinib treatment and, at last follow-up, retained Major Molecular Response (MMR).

The last one (n.4) relapsed after 4 years from allogeneic stem cell transplantation (SCT), achieved CCgR and complete MR after 6 months of imatinib therapy. Six years later, she lost MMR, started dasatinib and then nilotinib treatments, but she died in Partial CgR after the second allogeneic SCT.

## DISCUSSION

As is known, FISH analysis is an efficient tool to characterize Ph-neg rearrangements, because it allows to detect the occurrence of BCR/ABL rearrangement and to monitor the response to therapy [13].

To our knowledge, here we present the largest monocentric series of the of Ph-neg CML patients treated with TKIs therapies and we confirm their low frequency (3%).

In Ph-neg CML cases, the BCR/ABL fusion gene is often located on der(22) and rarely on der(9), indeed its presence on der(9) has been reported only in 24 Ph-neg CML patients [4–10]. Conversely, in our series 4/6 (66,7%) Ph-neg CML cases showed the rearrangement on der(9) and one displayed 2 fusion signals on both derivative chromosomes 9 and 22, as in the classic translocation.

Regarding the involving mechanisms, previous reports considered the cryptic insertion of chromosome 22, comprising 5′ BCR within the ABL gene or viceversa, more probable than two consecutive translocations. The insertion seems more likely, since it requires only two breaks at 22q11 and one at 9q34, or viceversa, instead of four breaks required in double consecutive translocations [5]. TCDF BCR/ABL FISH analysis allowed us to clarify the mechanisms by which the translocations may take place. We have hypothesized cryptic insertion in 5 cases and two-translocations mechanism in only one case. The observation of 4 different FISH pattern in 6 Ph-neg cases suggested high genetic heterogeneity in this subgroup of CML patients.

The prognostic significance of the occurrence of cryptic Ph translocations has been discussed in previous reports, but data regarding the outcome of Ph-neg CML patients treated with TKIs are limited. In 4 patients, the CgR by FISH analysis was achieved after a median time of 24.5 months from the start of imatinib (range 7-40 months): 3 patients are still in deep MR, while the other one lost MMR after 6 years of imatinib. Another patient achieved MMR when treated subsequently with nilotinib. Two patients had to change TKI because of resistance to imatinib and of relapse after SCT.

During imatinib treatment, a not responder patient showed duplication of the BCR/ABL rearrangement, confirming that this change is an event of clonal evolution. The duplication of rearrangement appeared as 2 fusion genes on both the homologous chromosomes 9, like in 2 other cases previously reported [10]. Similarly to our case, the 2 patients did not achieve CgR. The amplification of BCR/ABL rearrangement (as the duplication of Ph chromosome) has been always considered a mechanism of resistance to the therapy and, therefore, associated with adverse prognosis, like other additional chromosomal abnormalities [14, 15].

Moreover, deletions of a sizable portion of the der(9) have been described in 10-15% of CML patients. A recent paper asserted that deletions of der(9) do not influence the response and the outcome of CML in early CP treated with imatinib [16]. De Melo et al. [17] reported deletions of ABL or BCR regions in 21% of Ph-neg CML cases, concluding that the loss of genomic material is an effect of any DNA breakage event at chromosome 9q34 and 22q11 regardless of the mechanism of chromosomal rearrangement. In our cases, deletions occur in 2 patients (n.1 and 3): this rate is superior to that found in Ph+ patients [16], although we have to consider the low number of cases. However both our patients achieved MMR.

We have observed that 5 out of 6 Ph-neg CML patients achieved CCgR and/or MMR during TKIs treatment, also as second line of therapy. Clearly, the low number of cases and the heterogeneity of the therapy limit the analysis. We have to highlight that the adverse outcome of 2 patients could be related to their Sokal risk: intermediate and high, respectively. Moreover, the only patient with intermediate Sokal risk showed duplication of BCR/ABL rearrangement during the outcome.

In conclusion, most Ph-neg CML patients benefited from TKIs therapy and achieved CCgR and MMR, showing outcome similar to that of Ph+ CML patients.

Ph-neg rearrangements could be considered variant BCR/ABL rearrangements, although they involve only the chromosomes 9 and 22, differently to “real” variant translocations. As the latter [18], Ph-neg rearrangement does not influence the response to TKIs therapy. We confirm that Ph-neg CML patients do not constitute a “warning” category in imatinib era, in according to ELN 2013 recommendations [19, 20].

Only 2 Ph-neg CML patients failed TKIs therapy; in one a secondary change occurred, confirming that BCR/ABL duplication remains a mechanism of resistance to the therapy, also in TKI era.

## MATERIALS AND METHODS

### Patients

The clinical features of the Ph-neg patients are reported in Table 3. They were 5 males and 1 female, with a median age of 43.5 years (range 22-68); Sokal risk was low in 4 patients, intermediate in 1 patient and high in the last one; the median WBC count was 102.5*10^9^/l (range 32.9-283); the median PLT count was 324*10^9^/l (range 92-560); epatosplenomegaly was observed in all patients, but one (n.1).

**Table 3:** Clinical characteristics of the patients

Patients	Sex	Age^a^(years)	Sokal risk	Epato-splenomegaly	Count at diagnosis				Treatment	Lastdetail	Follow-up(months)
					WBC(10^9^/L)	PLT(10^9^/L)	BlastsBM (%)	Eo/Bso (%)			
1	M	22	low	No	52,3	398	1	3	hydroxyurea, imatinib	CCgR, MR^4^	156
2	M	68	intermediate	Yes	93	92	/	/	hydroxyurea, imatinib	died^b^	68
3	M	44	low	Yes	32,9	250	/	0,6	α-interferon, imatinib	CCgR, CMR, MR^5^	144
4	F	41	high	Yes	283	560	/	3	hydroxyurea, alloSCT, imatinib, dasatinib, nilotinib	died^c^	105
5	M	47	low	Yes	112	469	<5	0.75	imatinib	MMR, MR^4.5^	132
6	M	43	low	Yes	176	184	1	1,5	imatinib, nilotinib	MMR	lost

^a^at diagnosis; ^b^intestinal malignancy; ^c^in therapy with nilotinib;

Abbreviations: SCT: stem cell transplantation; CCgR= Complete Cytogenetic Response; MR= Molecular Response; CMR= Complete Molecular Response; MMR= Major Molecular Response.

Cytoreduction was started with hydroxyurea in 3 patients: later, 2 patients (n.1 and n.2) were treated with imatinib; meanwhile the other patient (n.4) underwent to allogeneic SCT, after 4 years she relapsed in accelerated phase and started imatinib therapy; then she was treated with dasatinib and afterwards with nilotinib. She died after second allogeneic SCT. Two other patients (n.5 and 6) were treated frontline with imatinb and the last one (n.3) started imatinib after α-IFN treatment.

The study is performed according to Good Clinical Practices and Declaration of Helsinki.

### Cytogenetics analysis

Cytogenetic studies were performed on bone marrow samples after short term (24 and/or 48 hours) cultures. Karyotypes were examined after GAW banding techniques. In each case, at least 20 metaphases were analysed and the karyotypes were described according to the criteria of the International System for Human Cytogenetic Nomenclature [21].

### FISH analysis

FISH was performed on bone marrow cells prepared according to standard cytogenetic techniques and using LSI BCR/ABL DCDF and LSI BCR/ABL + 9q34 TCDF translocation probes (Vysis Inc, Downers Grove, IL, USA) and ON BCR/ABL t(9;22), triple-color probe (Kreatech Biotechnology, Amsterdam, Netherlands). FISH was carried out according to the manufacturer’s instructions, with slight modifications. FISH analysis was performed at least on 200 nuclei and on metaphases to confirm the interphase FISH pattern and to localize the rearrangement.

Since at diagnosis FISH pattern of the most cases shows only 1 fusion signal by FISH analysis, CgR was defined by the rate of cells with 1 fusion signal. The value of false positive cut-off was calculated on 68 normal controls resulting in 10% (mean+3DS) [22].

### RT-PCR and RQ-PCR

Qualitative RT-PCR for BCR/ABL transcript was performed at diagnosis, in order to determine the type of transcript.

The RQ-PCR was performed to monitor the amount of BCR/ABL transcript during the therapy, using the ABIPRISM7700 Sequence Detector (Perkin Elmer, Faster City, CA). ABL was used as housekeeping gene to correct for differences in RNA quality and/or RT efficacy. All experiments were performed in duplicate and the results were expressed as percent ratio to ABL. The BCR/ABL to ABL ratios were additionally multiplied by the conversion factor of the Bologna lab to set the results on the International Scale (IS). Samples yielding an ABL threshold cycle (Ct) greater than 30, which corresponded to fewer than 1,000 ABL transcript copies, were considered to have degraded RNA and were discarded.

The following criteria have been used to define the molecular response (MR) [19, 23]:

MR^4^= either detectable disease ≤0.01% BCR-ABL^IS^ or undetectable disease in cDNA with ≥10000 ABL1 or ≥24000 GUSB transcripts (numbers of ABL or GUSB transcripts in the same volume of cDNA used to test for BCR-ABL);

MR^4,5^= either detectable disease ≤0.0032% BCR-ABL^IS^ or undetectable disease in cDNA with ≥32000 ABL1 or ≥77000 GUSB transcripts;

MR^5^= either detectable disease ≤0.001% BCR-ABL^IS^ or undetectable disease in cDNA with ≥100000 ABL1 or ≥240000 GUSB transcripts.
